# Efficacy of alcohol/sugar aqueous biphasic system on partition of bovine serum albumin

**DOI:** 10.1186/s40643-021-00360-y

**Published:** 2021-01-21

**Authors:** Yin Hui Chow, Alagan Sahlini, Hui-Suan Ng, John Chi-Wei Lan

**Affiliations:** 1 School of Computer Science and Engineering, Faculty of Information and Technology, Taylor’s University Lakeside Campus, 47500 Subang Jaya, Selangor Malaysia; 2 Faculty of Applied Sciences, UCSI University, No. 1, Jalan Menara Gading, UCSI Heights, Cheras, 56000 Kuala Lumpur, Malaysia; 3 Biorefinery and Bioprocess Engineering Laboratory, Department of Chemical Engineering and Materials Science, Yuan Ze University, Chungli, Taoyuan, 320 Taiwan

**Keywords:** Aqueous biphasic system, Separation, Protein partition, Alcohol, Carbohydrate

## Abstract

The efficacy of alcohol/sugar aqueous biphasic (ABS) system on protein extraction was investigated. A model protein, bovine serum albumin (BSA), was adopted to evaluate the effects of types and concentration of phase-forming components, protein concentration, and system pH on the protein partition efficiency. The 1-propanol/maltose ABS exhibited an overall better partition efficiency of BSA to the alcohol-rich top phase. A maximum partition coefficient (*K*) of 20.01 ± 0.05 and recovery yield (*Y*) of 95.42% ± 0.01% of BSA were achieved with 35% (w/w) 1-propanol/22% (w/w) maltose ABS at pH 5.0 for 10% (w/w) BSA load. The *K* and *Y* of BSA in 1-propanol/maltose ABS was slightly improved with the addition of 3% (w/w) of ionic liquid, 1-butyl-3-methylimidazolium bromide ([Bmim]Br) as the adjuvant that could provide protein stabilizing effect. The Fourier Transform Infrared Spectrum (FTIR) analysis revealed that the protein structure remained unaltered upon the separation process.

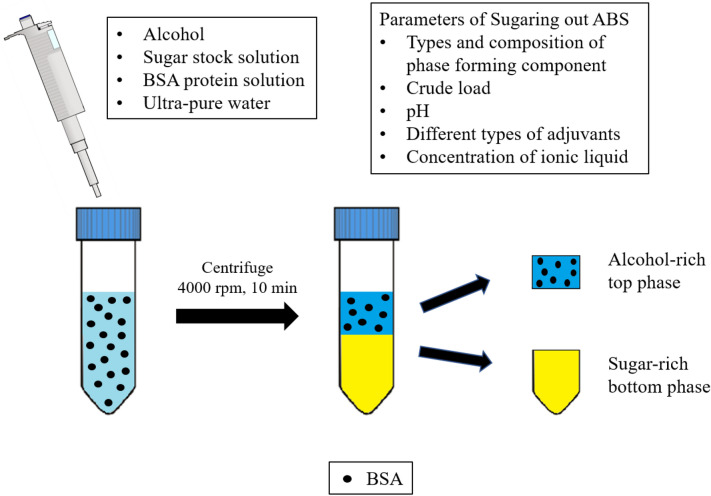

## Introduction

Aqueous biphasic system (ABS) with high water content has emerged as an alternative cost-effective approach for protein separation and purification. ABS is formed by mixing two immiscible phase-forming solutions at a critical composition (Ng et al. 2011). The difference in the physicochemical properties and the interactions between the biomolecules and the phase-forming molecules results in differential partitioning and separation of the target biomolecules in the ABS. ABS offers several process advantages, such as low interfacial tension, short phase separation time, and low toxicity. Polymer/polymer ABS, polymer/salt ABS, and alcohol/salt ABS have been widely applied in protein extraction (Ng et al. 2011). Nevertheless, the industrial-scale applications of polymer-based ABS are limited because of the high viscosity of polymer used and the high cost associated with the recycling of phase-forming components (Ng et al. 2012). Moreover, the use of high charge density salts may cause environmental issues and denaturation of targeted protein due to its high ionic strength or alkalinity.

The substitution of conventional salts with carbohydrates could potentially create a more biocompatible separation environment for the biomolecules (de Brito Cardoso et al. 2013). Carbohydrates are polyhydroxy aldehydes or ketones that encompass a broad range of organic compounds. It can be divided into two large groups: simple sugars (i.e. monosaccharides) and compound sugars (i.e. oligosaccharides and polysaccharides). Sugars are electrically neutral, non-toxic, biodegradable, and highly hydrophilic (Sadeghi et al. 2016). Sugars possess several hydroxyl groups with dual donor/acceptor characteristics that can participate in hydrogen bonding, thereby exerting sugaring-out effect (i.e., also known as soluting-out effect) inherently (de Brito Cardoso et al. 2013). Rising attention has been given to the application of carbohydrates as a sugaring-out agent to form polymer/sugar ABS (Sadeghi et al. 2016), acetonitrile/sugar ABS (de Brito Cardoso et al. 2013), ionic liquid (IL)/sugar ABS (Quental et al. 2018), and alcohol/sugar ABS (Ebrahimi and Sadeghi 2018). The viscosity of alcohol is lower when compared to polymers and ILs, allowing more rapid phase separation and efficient mass transfer of biomolecules from one phase to another (Wang et al. 2010). Considering these advantages, alcohol/sugar ABS could potentially serve as a low-cost and sustainable extraction platform for the recovery and separation of biomolecules. Although the phase composition and properties of alcohol/sugar ABS have been characterized (Ebrahimi and Sadeghi 2018), the separation efficiency of the alcohol/sugar ABS and the stability of the biomolecules after extraction remain superficial to date.

This study aims to investigate the partition efficiency of protein in alcohol/sugar ABS composed of short-chain aliphatic alcohols (1-propanol and 2-propanol) and different sugars (glucose, sucrose, and maltose). Carbohydrates, such as glucose, sucrose, and maltose, are low-cost sugars that can be easily derived from natural sources and are widely used in the food industries. Longer chain alcohol (e.g. butanol) has higher protein destabilizing power than the shorter and branched one (e.g. methanol, ethanol, and 1-propanol) and thus was not investigated in this study (Miyawaki and Tatsuno 2011). Serum albumin is the most abundant protein in vertebrates’ body fluid. It has numerous vital physiological functions. Bovine serum albumin (BSA), which has 76% structural similarity with human serum albumin (HSA), has been extensively applied as the model protein in various extraction and purification studies (Pereira et al. 2015). Hence, BSA was employed as the model protein to evaluate the efficacy of alcohol/sugar ABS on the partition of protein in this study.

ILs are often known as designer solvents due to their flexibility and tunability. These greener solvents are generally non-flammable, chemically stable, and possess high dissolving capacity and good extractability. Besides its application as phase-forming components, recent studies have shown that smaller amounts (≤ 5% (w/w)) of ILs can be added as adjuvants into polymer/salt ABS (Neves et al. 2019; Aziz et al. 2017) and alcohol/salt (Ran et al. 2019) to enhance the partition efficiency of biomolecules to one of the phases. In this study, the effects of the addition of neutral salts and ILs as adjuvants on the partition efficiency of BSA in alcohol/sugar ABS were examined. The stability of BSA upon the ABS separation process was also assessed with Fourier Transform Infrared Spectrum (FTIR) analysis.

## Materials and methods

### Materials

Lyophilized BSA (essentially globulin and fatty acid free, ≥ 99% pure) was purchased from Sigma-Aldrich (St. Louis, MO, USA). Ethanol absolute 99.8% (C_2_H_5_OH), 1-propanol (C_3_H_8_O) absolute 99.5%, and 2-propanol absolute 99.5% were obtained from VWR Chemicals (Fontenay-Sous-Bois, France). D-(+)-glucose (≥ 99.0%), sucrose (≥ 98.0%), and D-(+)-maltose monohydrate (≥ 99.0%) were obtained from Merck (Darmstadt, Germany). Pierce BCA protein assay kit was sourced from Thermo Scientific (Rockford, United States of America).

### Determination of binodal curves

The binodal curves were constructed according to the turbidimetric titration method using different concentrations of alcohol (i.e. 1-propanol and 2-propanol) and sugar (i.e. glucose, maltose, and sucrose) (Johansson et al. 2011). A mixture containing known concentrations of alcohol and sugar stock solution was weighed and titrated dropwise with an appropriate amount of ultrapure water until a single-phase solution was formed. The resultant mixture was centrifuged at 4000*g* for 10 min to ensure a single-phase system was formed. The final weight of the system was measured to determine the amount of ultrapure water added.

### Protein partitioning in alcohol/sugar ABS

The protein partitioning experiment was performed at room temperature (25 °C). ABS with a final mass of 5.0 g was prepared in a 15-mL centrifuge tube by mixing appropriate amounts of 100% (w/w) alcohol (i.e. 1-propanol and 2-propanol), 60% (w/w) stock solution of sugar (i.e. glucose, maltose, and sucrose), ultrapure water, and 5% (w/w) of 20 mg/mL of BSA protein solution unless otherwise stated (Ng et al. 2018). The final protein concentration in ABS was approximately equivalent to the BSA concentration (ca. 1 mg/mL) tested in other works and thus could provide the possibility for comparison of protein partition efficiency in various types of ABS. Subsequently, the mixture was centrifuged at 4000*g* for 10 min to ensure total phase separation was attained. The volume of each phase was recorded. Sample was collected separately from each phase to quantify the BSA concentration. All ABS partitioning experiments were performed in triplicate.

### Determination of protein content using bicinchoninic acid (BCA) assay

The concentration of BSA in each phase was determined using the bicinchoninic acid (BCA) assay (Ng et al. 2018). To each of the 25 µL of phase sample solution, 200 µL of working reagent was added. The resultant mixture was mixed thoroughly for 30 s and incubated at 37 °C for 30 min. Next, the absorbance of the resultant mixture was measured at 562 nm against the blank ABS phase sample solution which was prepared in parallel to eliminate any possible interferences. The calibration curve was constructed using BSA standard solutions diluted to the working range of 25–2000 µg/mL.

### Fourier Transform Infrared Spectrum (FTIR) analysis

The Fourier-Transform Infrared Spectrometer (FTIR) analysis was conducted to examine the protein conformation and stability of BSA before and after the ABS extraction (Pereira et al. 2015). The FT-IR spectra (4 cm^−1^ resolution, 16 scans) were recorded using the Perkin Elmer Spectrum 100 FT-IR spectrometer (BioTek, Winooski, VT, USA) in the wavelength ranging from 450 to 4000 cm^−1^ to locate the main functional group.

### Determination of protein partition efficiency

The volume ratio (*V*_*R*_) was expressed as the ratio of the volume of the top phase (*V*_*T*_) to the volume of the bottom phase (*V*_*B*_):

1 $$ V_{R}  = \frac{{V_{T} }}{{V_{B} }} . $$

The partition coefficient (*K*) of BSA was calculated as the ratio of the concentration of BSA at the top phase (*C*_*T*_) to the concentration of BSA at the bottom phase (*C*_*B*_) (Eq. 2):

2 $$ K = \frac{{C_{T} }}{{C_{B} }} . $$

The recovery yield (*Y*) was used as a parameter to evaluate the percentage of the amount of BSA partitioned between the top phase and the total mixture. The *Y* was calculated as a function of *V*_*R*_ and *K* according to (Eq. 3) (Ng et al. 2019):

3 $$ Y = \frac{100}{{1 + \frac{1}{{V_{R} \cdot K}}}} . $$

## Results and discussion

### Binodal curves of sugaring-out assisted ABS

**Fig. 1:**
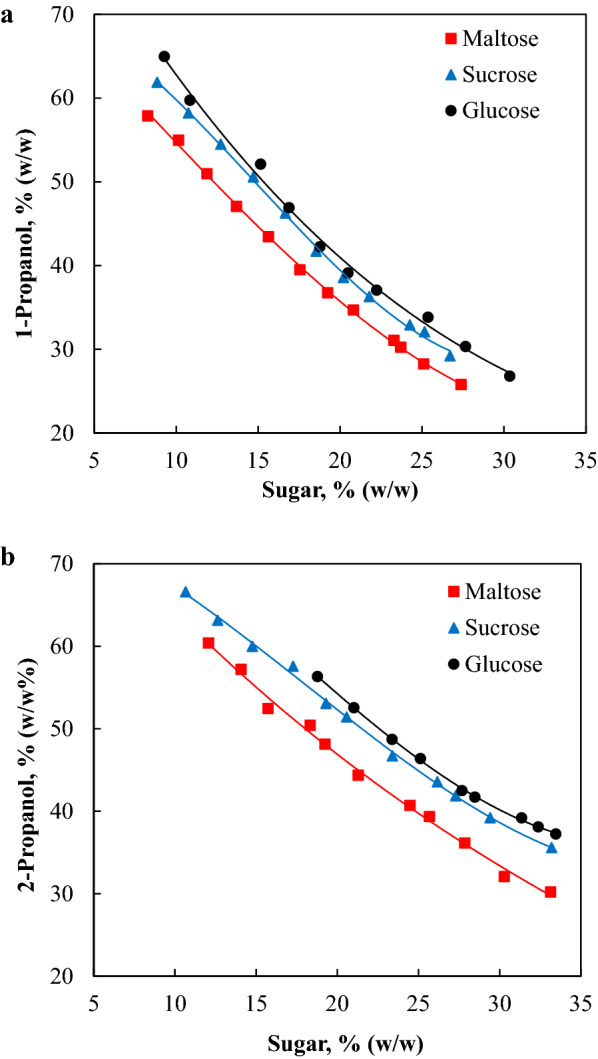
Binodal curves of alcohol/sugar ABS. **a** Binodal curves of 1-propanol/sugar ABS and **b** Binodal curves of 2-propanol/sugar ABS. The solid lines of the binodal curves were obtained by connecting the sufficient points experimentally

Figure 1a and b depicts the binodal curves of 1-propanol/sugar ABS and 2-propanol/sugar ABS, respectively. The biphasic region of 1-propanol/sugar ABS was larger than that of 2-propanol/sugar ABS. This trend indicates that 1-propanol exhibits greater phase-forming ability compared to 2-propanol. According to previous report, the octanol/water partition coefficient (*K*_*ow*_) value of 1-propanol is 1.78 and the *K*_*ow*_ of 2-propanol is 1.12 (Ebrahimi and Sadeghi 2018). The higher *K*_*ow*_ value of 1-propanol isomer suggests that it has higher hydrophobicity and lower solubility in water and polar substances. Furthermore, the aqueous 1-propanol solution has a higher surface tension compared to the aqueous 2-propanol solution, thereby facilitating more effective phase separation (Erfani et al. 2019). Thus, a lower concentration of 1-propanol is sufficient to form ABS with sugar. Ethanol was also investigated as a potential phase-forming component for the formation of alcohol/sugar ABS. Several systems constituted with various concentrations of ethanol and sugar were examined. However, no phase formation was observed. This could be due to the relatively close hydrophilicity degree between the ethanol and the sugars investigated (Ebrahimi and Sadeghi 2018). Moreover, precipitation was observed in the mixture which contained a high concentration of ethanol and a low concentration of sugar. Thus, ethanol and sugars are not suitable candidates to form stable alcohol/sugar ABS.

**Fig. 2:**
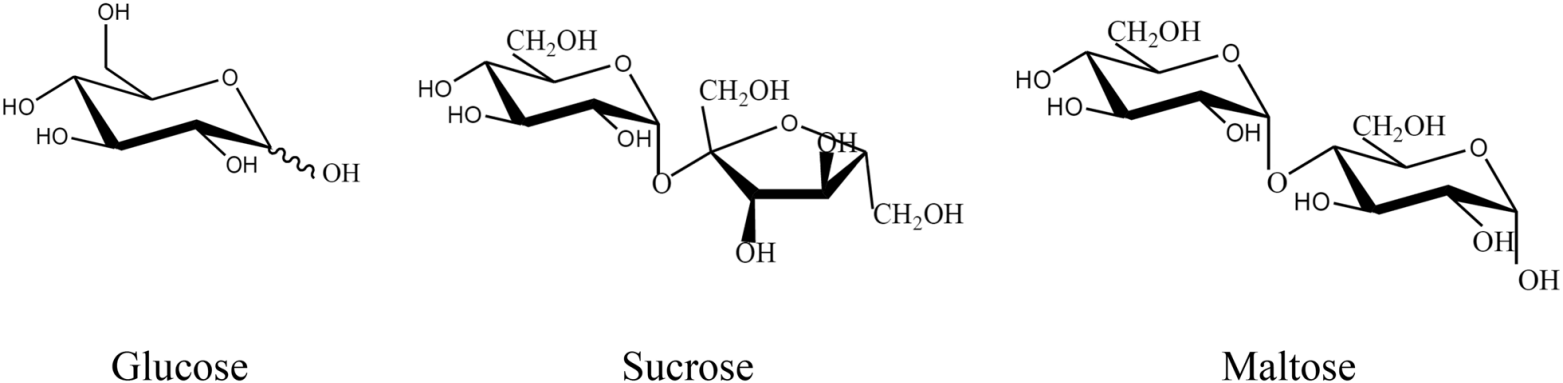
Molecular structure of sugars used to form the alcohol/sugar ABS

Figure 1a shows that the binodal curve of 1-propanol/maltose ABS was closer to the axes compared to the binodal curves of 1-propanol/glucose ABS and 1-propanol/sucrose ABS. A similar trend was observed for 2-propanol/sugar ABS (Fig. 1b), indicating that maltose has higher phase-forming ability compared to sucrose and glucose. The trend was in agreement with the sugaring-out ability and hydrophilicity of the sugars investigated. It has been reported that the ability of these sugars in sugaring-out ionic liquids (Ferreira et al. 2016), polymers (Sadeghi et al. 2016), and alcohols (Ebrahimi and Sadeghi 2018) for the formation of ABS increased in the order of glucose < sucrose < maltose. As shown in Fig. 2, the disaccharide maltose possesses a higher number of equatorial hydroxyl groups compared to sucrose and glucose, thereby leading to a higher affinity of maltose for water and stronger sugaring-out ability (Freire et al. 2011; Ebrahimi and Sadeghi 2016). Thus, the amount of maltose required to form ABS with 1-propanol and 2-propanol was lower compared to sucrose and glucose as shown in Fig. 1.

### Effect of types and concentrations of phase-forming components on the partition efficiency of BSA

The partitioning behaviour of BSA in 1-propanol/sugar ABS and 2-propanol/sugar ABS was investigated to evaluate the effect of various types and concentrations of phase-forming components on the partition efficiency of BSA. The *K* and recovery yield (*Y*) of BSA in the alcohol-rich top phase are shown in Table 1. The investigated systems were chosen based on their relative position in the biphasic region which gave a *V*_*R*_ of 1.0 at equilibrium.

**Table 1:** Effect of types and concentration of phase-forming components on the partition efficiency of BSA with propanol/sugar ABS

Types of ABS	Concentration of sugar, % (w/w)	Concentration of alcohol, % (w/w)	*K*	Recovery yield, %
1-Propanol/Maltose	22	35	6.03 ± 0.17	87.84 ± 0.61
	23	37	4.40 ± 0.43	81.70 ± 1.88
	24	38	2.57 ± 0.46	72.58 ± 3.58
	25	39	1.74 ± 0.14	64.39 ± 1.81
	26	39	0.90 ± 0.25	49.40 ± 4.79
1-Propanol/Sucrose	26	33	1.17 ± 0.07	63.61 ± 1.48
	27	34	1.26 ± 0.11	56.71 ± 2.11
	28	35	1.36 ± 0.15	62.86 ± 2.64
	29	36	0.93 ± 0.10	48.21 ± 2.72
	30	37	0.72 ± 0.08	42.33 ± 3.32
1-Propanol/Glucose	24	36	1.14 ± 0.08	50.42 ± 2.10
	25	38	1.36 ± 0.05	58.11 ± 1.48
	26	39	1.01 ± 0.08	50.76 ± 2.58
	27	40	0.59 ± 0.02	38.04 ± 0.71
	28	41	0.38 ± 0.09	30.75 ± 4.91
2-Propanol/Maltose	31	32	2.41 ± 0.39	70.51 ± 3.35
	32	33	1.19 ± 0.19	54.19 ± 3.88
	33	34	0.85 ± 0.03	42.10 ± 0.92
	34	35	0.76 ± 0.14	43.06 ± 4.63
	35	36	0.33 ± 0.03	23.38 ± 1.66
2-Propanol/Sucrose	33	38	1.01 ± 0.03	48.13 ± 0.84
	34	39	0.75 ± 0.02	43.02 ± 0.60
	35	40	0.74 ± 0.04	42.46 ± 1.19
	36	41	0.22 ± 0.05	17.71 ± 3.15
	37	41.5	0.08 ± 0.03	7.42 ± 2.90
2-Propanol/Glucose	31	39	0.82 ± 0.07	44.95 ± 2.00
	32	40	0.59 ± 0.00	39.84 ± 0.10
	33	41	0.35 ± 0.05	27.38 ± 2.70
	34	42	0.32 ± 0.04	25.82 ± 2.51
	35	43	0.48 ± 0.02	34.33 ± 1.10

Alcohol/sugar ABSs comprised of different phase-forming component concentration were selected for investigation based on their relative position in the biphasic region which gave a *V*_*R*_ of 1.0 at equilibrium

Results in Table 1 show that the 1-propanol/sugar ABS demonstrated better partition efficiency of BSA than 2-propanol/sugar ABSs. This could be attributed to the relatively higher hydrophobicity of 1-propanol compared to 2-propanol that promotes the partition of BSA towards the alcohol-rich top phase of 1-propanol/sugar ABS (Ng et al. 2018). Furthermore, 1-propanol/sugar ABSs with maltose-rich bottom phase gave an overall better protein partition efficiency compared to 1-propanol/sucrose ABS and 1-propanol/glucose ABS. A similar trend was observed in the 2-propanol/sugar ABS. The effect of types of sugar on the protein partition efficiency could be explained by the sugaring-out ability of sugars and their hydration extensions (Ebrahimi and Sadeghi 2018). The intensity of the sugaring-out effect depends on the differential position and the number of hydroxyl groups on the pyranose ring. Equatorial hydroxyl groups possess greater hydration potential than the axial ones as the former could interact with water molecules and form long-lived hydration structures (Ng et al. 2012; Sadeghi et al. 2016). For glucose, its C-2, C-3, and C-4 hydroxyl groups are all in the equatorial positions (Fig. 2). Maltose and sucrose possess the same number of hydroxyl group. Maltose is composed of two glucose monomers, whereas sucrose is composed of a glucose monomer and a fructose monomer. As such, maltose which consists of two six-membered pyranose rings is more easily hydrated than sucrose (Freire et al. 2011). In other words, maltose which has a higher number of equatorial hydroxyl groups could exert a stronger sugaring-out effect than sucrose and glucose, thereby improving the transfer of BSA to the alcohol-rich top phase.

Based on Table 1, the optimal condition for BSA extraction was observed in 35% (w/w) 1-propanol/22% (w/w) maltose ABS, which had a maximum *K* of 6.03 ± 0.17 and *Y* of 87.84% ± 0.61%. These results indicate that the partition efficiency of BSA is better at lower concentrations of 1-propanol and maltose. As the alcohol-rich top phase is more hydrophobic compared to the sugar-rich bottom phase, the hydrophobic interactions between the BSA and 1-propanol-rich phase are probably the main driving force for the preferential partition of BSA to the alcohol-rich top phase (Ng et al. 2018).

In general, the increase in alcohol and sugar concentrations were unfavourable for the partition of BSA in 1-propanol/sugar ABSs and 2-propanol/sugar ABSs. Further increase of alcohol and sugar concentrations reduced the *K* to below unity (*K* < 1) and decreased the *Y* to below 50% for all the investigated alcohol/sugar ABSs. This decrease in partition efficiency could be attributed to the gradual dehydration of both aqueous phases as the phase-forming components’ concentration increases, but to a stronger extent in the alcohol-rich top phase (Ebrahimi and Sadeghi 2018). This dehydration effect resulted in insufficient free water molecules to solubilize the protein in the alcohol-rich top phase and exclusion of protein to the bottom phase, thereby resulting in the decrease of protein partition efficiency (Ooi et al. 2009). Therefore, 35% (w/w) 1-propanol/22% (w/w) maltose ABS which has the highest protein partition efficiency was selected for further studies.

### Effect of BSA amount added to the ABS on the partition efficiency of BSA

**Fig. 3:**
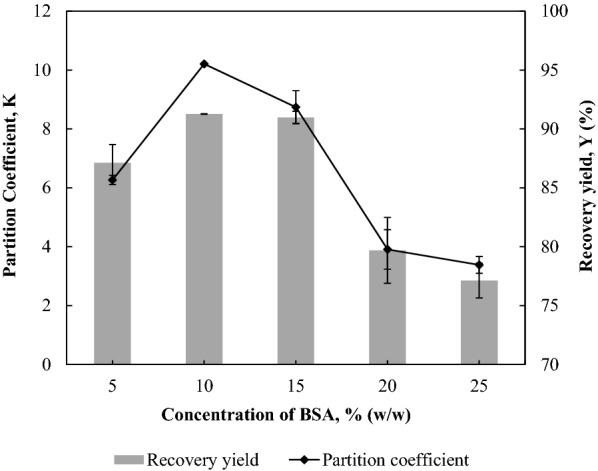
Effect of amount of BSA added to the ABS on the partition efficiency of BSA. The amount of BSA was varied at a range of 5–25% (w/w) to evaluate the effect of protein concentration on the *K* and *Y* of BSA in the 35% (w/w) 1-propanol/22% (w/w) maltose ABS. The results are expressed as mean ± standard deviation of triplicate readings

The amount of BSA added into the ABS was varied at a range of 5–25% (w/w) to evaluate the partition efficiency of BSA in the 35% (w/w) 1-propanol/22% (w/w) maltose ABS (Fig. 3). When the amount of protein added to the system was increased from 5% (w/w) to 10% (w/w), the partition coefficient increased from 6.27 ± 0.16 to 10.21 ± 0.03 and the recovery yield increased from 87.12% ± 1.55% to 91.27% ± 0.02%. It was observed that the increase in protein amount from 5% (w/w) to 25% (w/w) resulted in a decrease of *V*_*R*_ by 8.7%. With the decrease in *V*_*R*_, the free volume which is available in the top phase to accommodate a higher amount of protein is greatly reduced (Ooi et al. 2009). Hence, the protein partition efficiency decreased to a minimum *K* of 3.38 ± 0.28 and *Y* of 77.12% ± 1.49% when the protein amount was increased to 25% (w/w). The increase in the amount of BSA beyond 25% (w/w) is not feasible due to the increase in the tendency of protein precipitation at the interface as a result of phase saturation (Ng et al. 2014). Thus, 10% (w/w) of BSA solution which exhibited the highest protein partition efficiency was selected to further investigate the effect of pH on the partitioning behaviour of BSA in the 1-propanol/maltose ABS.

### Effect of pH on the partition efficiency of BSA

The pH of an ABS can be adjusted to steer and enhance the partition of protein to the targeted phase. In this study, the pH of the 35% (w/w) 1-propanol/22% (w/w) maltose ABS containing 10% (w/w) BSA was varied between pH 3.0 and pH 8.0 to investigate the effect of pH on the partition efficiency of BSA. This pH range was selected by considering the isoelectric point (pI = 4.8) of BSA and its relative stability at pH 5.0–8.0 (Chow et al. 2015). The phase system was mixed well while adjusting the pH with the addition of 1.0 M of sulphuric acid or 1.0 M sodium hydroxide. The change in the volume of both phases was negligible with the increase in pH.

**Fig. 4:**
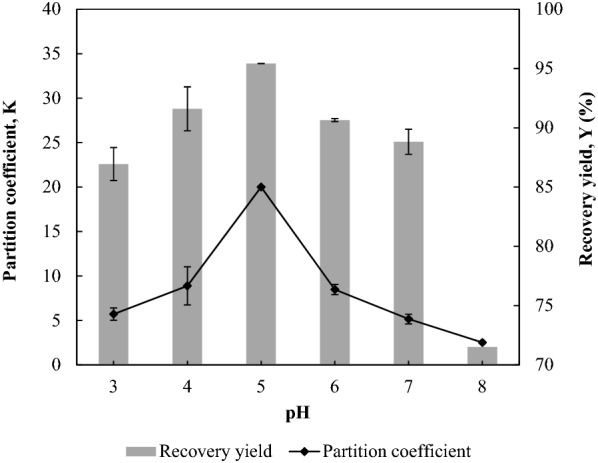
Effect of pH on the partition efficiency of BSA. The pH of the 35% (w/w) 1-propanol/22% (w/w) maltose ABS with 10% (w/w) BSA was varied between pH 3.0 and pH 8.0 to investigate the effect of pH on the *K* and *Y* of BSA. The results are expressed as mean ± standard deviation of triplicate readings

As shown in Fig. 4, the pH exerted a significant impact on the protein partition efficiency. When the pH was increased from pH 3.0 to pH 5.0, the partition efficiency of BSA increased remarkably from *K* of 5.71 ± 0.7 and *Y* of 86.94% ± 1.39% at pH 3.0 to maximum *K* of 20.01 ± 0.05 and *Y* of 95.42% ± 0.01% at pH 5.0. When the pH was increased to pH 5.0, approaching the pI of BSA, the net surface charge of BSA was close to zero (Chow et al. 2015). Thus, the hydrophobic interaction between the BSA and 1-propanol molecules was intensified at this pH, facilitating the partition of more BSA to the alcohol-rich top phase (Ng et al. 2018). Thereafter, the protein partition efficiency decreased significantly when the pH was raised to pH 8.0. At pH 8.0, the *K* and *Y* of BSA dropped to a minimum of 2.52 ± 0.27 and 71.50% ± 2.17%, respectively. No protein precipitation was observed for all the investigated pH values. As proteins prone to denature at high pH values, operating the ABS above pH 8.0 would not be favourable for effective protein recovery (Chow et al. 2015).

### Effect of types of adjuvants on the partition efficiency of BSA

**Fig. 5:**
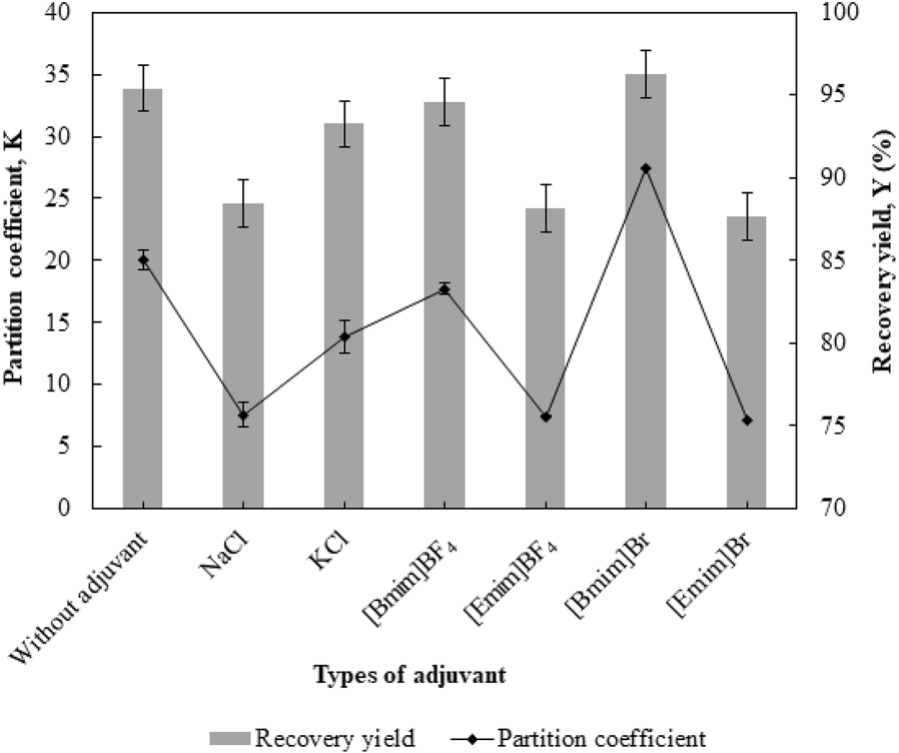
Effect of types of adjuvants on the partition efficiency of BSA. Neutral salts, such as NaCl and KCl, and ILs, such as [Bmim]BF_4_, [Emim]BF_4_, [Bmim]Br and [Emim]Br, were added at a fixed concentration of 1% (w/w) to the 35% (w/w) 1-propanol/22% (w/w) maltose ABS at pH 5.0 as adjuvants to evaluate the effect of types of adjuvant on the *K* and *Y* of BSA. The results are expressed as mean ± standard deviation of triplicate readings

The effects of the addition of neutral salts (sodium chloride (NaCl) and potassium chloride (KCl)) or ILs (1-butyl-3-methylimidazolium tetrafluoroborate, [Bmim]BF_4_; 1-ethyl-3-methylimidazolium tetrafluoroborate, [Emim]BF_4_; 1-butyl-3-methylimidazolium bromide, [Bmim]Br; and 1-ethyl-3methylimidazolium bromide, [Emim]Br) on the *K* and *Y* of BSA in the 35% (w/w) 1-propanol/22% (w/w) maltose ABS at pH 5.0 were investigated (Fig. 5). The concentration of adjuvant added into the ABS was fixed at 1% (w/w) and kept at a minimal value to ensure negligible change in phase composition and *V*_*R*_.

According to the Hofmeister series, the salting-out effect is more pronounced for KCl than NaCl (Wan et al. 2018). Thus, higher *K* (13.86 ± 1.03) and *Y* (93.26% ± 0.47%) were obtained in the 35% (w/w) 1-propanol/22% (w/w) maltose ABS which was added with KCl compared to *K* (7.57 ± 0.78) and *Y* (88.48% ± 1.34%) obtained with the addition of NaCl. Nevertheless, the *K* and *Y* values attained with the addition of these univalent neutral salts were lower than that without the addition of adjuvants, indicating that the presence of KCl and NaCl has a negative impact on the partition efficiency of BSA in the alcohol/sugar ABS.

Among the ILs investigated, only [Bmim]Br exhibited a positive impact on the protein partition efficiency. When compared to the alcohol/sugar ABS without adjuvants, the addition of [Bmim]BF_4_ showed insignificant changes in the protein partition efficiency, while the addition of [Emim]BF_4_ and [Emim]Br reduced the *K* and *Y* significantly by more than 60% and 8%, respectively. When the effect of IL’s cation on the protein partition efficiency was compared, the *K* and *Y* values of BSA for ILs which share the same type of anion increase in the following orders: [Emim]BF_4_ < [Bmim]BF_4_ and [Emim]Br < [Bmim]Br. This phenomenon could be attributed to the alkyl chain length of IL’s cation. Longer alkyl chain length increases the hydrophobicity of ILs and enhances the molecular interaction between BSA and IL, thereby promoting the partition of the BSA in the top phase containing 1-propanol and IL (Ran et al. 2019). For the same alkyl chain length, [Bmim]Br exhibited higher *K* and *Y* values compared to [Bmim]BF_4_. This variation in the protein partition efficiency is in accordance with the chaotropic order of the IL anions (${\text{BF}}_{4}^{ - }$ > ${\text{Br}}^{ - }$), whereby the chaotropic ${\text{BF}}_{4}^{ - }$ which has a higher tendency in unfolding the protein and destabilizing the hydrophobic aggregates will increase the dissolution of protein in the sugar-rich bottom phase (Ran et al. 2019). Thus, the addition of tetrafluoroborate-based IL decreased the partition efficiency of protein in the alcohol/sugar ABS. Comparing to the ABS without adjuvant (*K* of 20.01 ± 0.05 and *Y* of 95.42% ± 0.01%), a slight increase in *K* (27.42 ± 0.02) and *Y* (96.27% ± 0.10%) were observed with the addition of [Bmim]Br. To further study the effect of addition of IL, [Bmim]Br was chosen to evaluate the effect of the concentration of IL on the partition efficiency of protein in the subsequent experiment.

### Effect of concentration of adjuvant [Bmim]Br on the partition efficiency of BSA

**Fig. 6:**
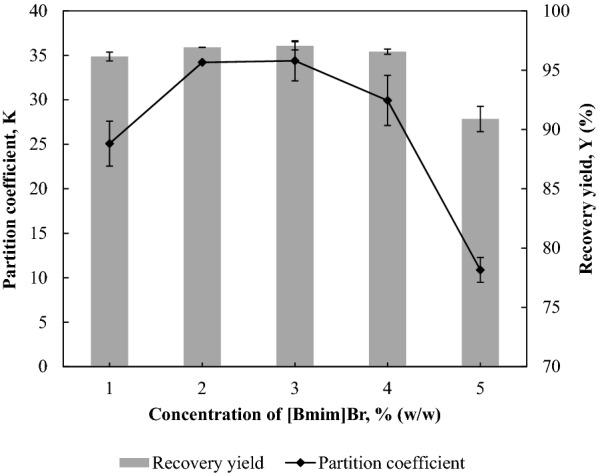
Effect of concentration of adjuvant [Bmim]Br on the partition efficiency of BSA. The effect of [Bmim]Br’s concentration ranging from 1.0% (w/w) to 5.0% (w/w) on the *K* and *Y* of BSA was evaluated. The results are expressed as mean ± standard deviation of triplicate readings

The effect of [Bmim]Br’s concentration on the partitioning behaviour of BSA was evaluated within the range of 1.0–5.0% (w/w), and the results are presented in Fig. 6. When the concentration of [Bmim]Br was increased to 3.0% (w/w), there was a slight increase in *Y* (97.05% ± 0.35%) by 1.63% and a marked increase in *K* (34.39 ± 2.26) by 72% compared to the system without adjuvant. This slight increase in protein partition efficiency indicates that the addition of IL could serve to enhance the affinity of the 1-propanol-rich phase to the protein (Ran et al. 2019). The protein partition efficiency decreased thereafter to *K* of 10.08 ± 1.4 and *Y* of 90.88% ± 1.06% at 5% (w/w) [Bmim]Br. This decrease in protein partition efficiency is probably caused by the change in protein stability at a high concentration of IL (Hadzir et al. 2016).

### Stability of BSA in 1-propanol/maltose ABS

**Fig. 7:**
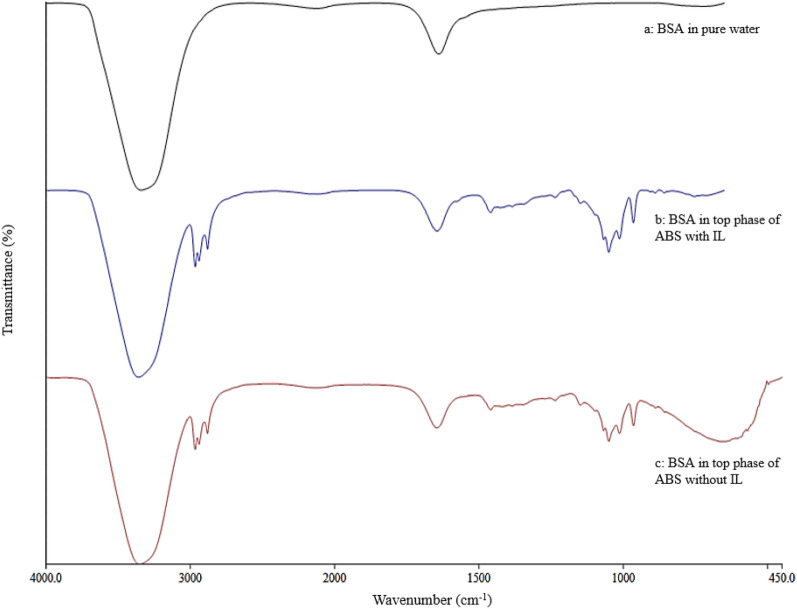
FTIR spectra of BSA in various solution. **a** BSA in pure water; **b** BSA partitioned in the alcohol-rich top phase of the 35% (w/w) 1-propanol/22% (w/w) maltose ABS at pH 5 and added with 3% (w/w) [Bmim]Br; **c** BSA partitioned in the alcohol-rich top phase of the 35% (w/w) 1-propanol/22% (w/w) maltose ABS at pH 5

The FTIR spectra of pure BSA and BSA partitioned in the alcohol-rich top phase of the 35% (w/w) 1-propanol/22% (w/w) maltose ABS at pH 5 with and without the addition of 3% (w/w) [Bmim]Br IL are illustrated in Fig. 7. FTIR spectra could provide useful information for determining the protein secondary structure based on the presence of energy absorption bands of specific functional groups. Proteins are made up of many amino acids that are joined to one another by amide bonds. The C=O bonds of the amide are involved in hydrogen bonding between different elements of the protein secondary structure. The amide I band which falls between 1600 and 17,000 cm^−1^ is associated with the C=O stretching and ring stretching vibrations of the amide functional group (Pei et al. 2009). As the most sensitive spectral region to the secondary structure of protein, this characteristic amide I absorption band is often used as a structural probe to determine the structural properties of protein.

In Fig. 7, the amide I absorption band of the pure BSA falls between 1600 and 17,000 cm^−1^. For the spectra of BSA separated to the top phase of 1-propanol/maltose ABS with and without IL, the amide I absorption band was readily identifiable in the same region (i.e., 1600–17,000 cm^−1^) without any shift caused by the presence of the ABS’s phase-forming components and the added adjuvant. These observations demonstrated that the spatial structure of the BSA could be conserved in the phase solution of the 1-propanol/maltose ABS.

## Conclusion

This work examined the partition efficiency of BSA in ABSs composed of various alcohols (i.e., 1-propanol and 2-propanol) and sugars (i.e., glucose, sucrose, and maltose). The types and concentration of phase-forming components, protein concentration, and pH have shown significant effect on the partition efficiency of protein. At low concentrations of alcohol and sugar, the BSA partitioned preferentially to the alcohol-rich top phase of the alcohol/sugar ABS. Maximum protein partition efficiency with *K* of 20.01 ± 0.05 and *Y* of 95.42% ± 0.01% could be achieved in the 35% 1-propanol/22% (w/w) maltose ABS at pH 5.0 which contained 10% (w/w) BSA. The FTIR analysis showed that the BSA structure was conserved in the 1-propanol/maltose ABS. A slight improvement in the partition efficiency of BSA was observed with the addition of 3% (w/w) of [Bmim]Br as adjuvant into the ABS. However, considering the additional processing cost incurred for large-scale implementation, the addition of IL is not recommended. Comparing to the expensive IL/sugar ABS, highly viscous polymer/sugar ABS, and the use of inorganic salt in polymer/salt ABS which could lead to corrosion of equipment and environmental pollution, the alcohol/sugar ABS could serve as a green and cost-effective alternative for the separation and purification protein.

## Data Availability

All the datasets obtained in this study are included in this article.

## Abbreviations

ABS: Aqueous biphasic system
(Bmim)BF_4_: 1-Butyl-3-methylimidazolium tetrafluoroborate
(Bmim)Br: 1-Butyl-3-methylimidazolium bromide
BCA: Bicinchoninic acid
BSA: Bovine serum albumin
C: Carbon
(Emim)BF_4_: 1-Ethyl-3-methylimidazolium tetrafluoroborate
(Emim)Br: 1-Ethyl-3methylimidazolium bromide
FTIR: Fourier Transform Infrared Spectrum
HSA: Human serum albumin
IL: Ionic liquids
K: Partition coefficient
KCl: Potassium chloride
NaCl: Sodium chloride
O: Oxygen
V_R_: Volume ratio
Y: Yield
